# Effects of a collaborative and gamified online learning methodology on class and test emotions

**DOI:** 10.1007/s10639-023-11879-2

**Published:** 2023-05-24

**Authors:** Javier Perez-Aranda, Samuel Medina-Claros, Ricardo Urrestarazu-Capellán

**Affiliations:** 1 Department of Economics and Business Management, University of Malaga, Campus El Ejido, E-29071 Malaga, Spain; 2 Department of Applied Economics (Public Finance, Economic Policy and Political Economy), University of Malaga, Campus El Ejido, E-29071 Malaga, Spain

**Keywords:** Gamification and collaborative learning, Online learning, Achievement emotions questionnaire, Attitude, social interactions, and participation, Higher education

## Abstract

This study examines the influence of students’ individual attitude and social interactions on participation in collaborative and gamified online learning activities, as well as the influence of participating in those activities on students’ online class- and test-related emotions. Based on a sample of 301 first year Economics and Law university students and using the Partial Least Squares-Structural Equation Modelling approach, all the relationships among first-order and second-order constructs included in the model are validated. The results support all the hypotheses studied, confirming the positive relationship that both students’ individual attitude and social interactions have on participation in collaborative and gamified online learning activities. The results also show that participating in those activities is positively related with class- and test-related emotions. The main contribution of the study is the validation of the effect of collaborative and gamified online learning on university students’ emotional well-being through the analysis of their attitude and social interactions. Moreover, this is the first time in the specialised learning literature that students’ attitude is considered as a second-order construct operationalised by three factors: the perceived usefulness that this digital resource brings to the students, the entertainment that this digital resource brings to the students, and the predisposition to use this digital resource among all those available in online training. Our findings aim to shed light for educators when preparing and designing computer mediated and online teaching programs that seek to generate positive emotions as a motivation for students.

## Introduction

Computer mediated learning and online learning are attracting educational specialists’ attention in higher education and have been described as one of the most effective and popular modes of instruction adopted by educators (Saqr & López-Pernas, 2021). Among other benefits, online learning has the capacity to adapt to a wide range of different learning methodologies. In a well-planned atmosphere, online methodologies might contribute to enhancing active and collaborative learning (Lai, 2021), students´ motivation (Radkowitsch et al., 2020), several aspects of general well-being (Johnson et al., 2016) as well as academic emotions (Cress et al., 2019). In contrast, some other authors who studied different online learning processes also showed that students’ attitude and motivation were poor (Zizka & Probst, 2022), their satisfaction was below expectations and dropout rates were high (Jiang et al., 2021), or reported a feeling of isolation (Rizvi & Nabi, 2021), stress and anxiety (Lemay et al., 2021).

A closer look at learning methodologies reveals that computer mediated online learning studies point to collaborative and gamified learning as inductors of positive outcomes. Furthermore, difficulties in generating collaborative learning in online environments have been reported (Sjølie et al., 2022), a circumstance that has become more evident during the COVID-19 pandemic, and it has been confirmed that perceived social support has the capacity to reduce the risk of psychological distress and student withdrawal (Tinajero et al., 2020). Therefore, in recent years attention has been drawn to the gap that exists in respect to the scaffolding and designing of better collaborative learning methodologies and how gamification in learning environments can affect physical, emotional, cognitive and social well-being (Melo et al., 2020; Schnaubert & Vogel, 2022). From a gamified and game-based perspective, the latest research has confirmed that game elements positively affect problem solving experiences (Dai et al., 2020), students’ engagement (Sinha et al., 2015), and formulation of emotions (Dondio et al., 2022).

The Control-Value theory of Achievement Emotions (Pekrun, 2006) is a comprehensive framework for exploring antecedents of students’ emotions. According to this theory, academic emotions are closely related to learning achievement and academic performance. They might help increase students’ intention to continue online learning and reduce dropout rates during a difficult period. However, the literature on learning methodologies, such as role-playing, combining computer based social interaction and its association with academic experiences and emotions is scarce (Järvelä & Rose, 2022). Even when some studies address the association of computer-supported collaborative learning with learning performance (Wu et al., 2021), no previous literature relates the use of these methodologies with other key aspects in students´ performance such as online class- and test-related experiences in which students´ emotions are essential (Roos et al., 2022).

Furthermore, higher university students face numerous classes, tests and evaluations during their academic life that are increasingly associated with anxiety and emotional problems (Putwain et al., 2010; Pekrun., 2023). In turn, these problematic situations are negatively associated with information processing and retrieval, self-esteem, anxiety and students’ general well-being, as well as increased social and emotional problems (Van Yperen, 2007; Melo et al., 2020; Cassady, 2022). In this context, research about the antecedents and effects of non-desirable emotions is crucial for the implementation of suitable prevention and intervention methodologies (von der Embse et al., 2018).

Hence, the objective of this study is to analyse whether students’ participation in Collaborative, Gamified and Online Learning (CGOL) activities is associated with positive emotions in connection with attending a class and with tests. Moreover, we examine if the students’ attitude as well as their social interactions predict their participation in those CGOL methodologies. Our empirical analysis is focused on first year undergraduate students, since their personal attitude and social relationships are particularly important due to the support they provide in stressful situations (Wilcox et al., 2005).

The significance and importance of our contribution to the problem situation is that we provide a feasible solution for educators and trainers who are facing the challenge of mitigating students’ negative emotions or generating positive experiences for them. Implementing the proposed collaborative and gamified online methodology will enable students participating in these activities to improve emotional and social well-being through their positive academic emotions.

This study contributes to the literature not only by focusing on outcomes of participation—construed as an active involvement—in the designed methodology but also on their antecedents. The participation outcomes included in the model are class- and test-related emotions. In respect to antecedents, the model includes the effect of social interaction and students´ attitude, which is measured as a higher order construct by (i) perceived usefulness, construed as the value or usefulness that this digital resource brings to the students (Boateng et al., 2016), (ii) entertainment, construed as the enjoyment that this digital resource brings to the students (Waiguny et al., 2012), and (iii) habit, construed as the predisposition to use this digital resource among all those available in online training (Greene et al., 2021).

## Literature review

### Collaborative and gamified online learning

Educational systems require a constant process of transformation and evolution enabling them to adapt to social, economic and technological changes of the environment. In some cases, this need to adapt has materialised in remote educational practices in which there is no direct personal contact with students, and interactions take place by means of Information and Communication Technologies.

When designing a teaching methodology, it is advisable to combine different methods as well as simulations referred to real and complex situations in order to foment more integrating thinking, which obliges students to assume an active participation through analysis and decision-making. For this study, collaborative learning and gamified learning methodologies, together with online learning practices, were jointly applied to offer benefits in aspects such as motivation or emotional and cognitive commitment. At present, these methods have been reinforced by incorporating new technological applications and digital methodologies to the educational process, successfully improving some of the features of collaborative and online education. Currently, these methods are closely linked to electronic and computing devices, in particular since the irruption of the COVID 19 pandemic in March 2020.

The first educational methodology included in our study is *collaborative learning* (CL), based on students’ joint and symmetrical active participation towards shared learning objectives by means of frequent and accessible communication (Nkhoma et al., 2017). Collaborative learning is the instruction method in which learners work together in small groups to achieve a common objective, independently of their performance levels. The main elements of all collaborative learning are: positive interdependence, interpersonal and small group skills, individual and group accountability, face-to-face promotive interaction, and group processing (Dillenbourg et al., 2009). With the inclusion of this methodology, we establish direct contact between first-year students and help them struggle with socialisation at a moment and in an academic year that presents many challenges in that respect.

The second methodology that was considered in the study is *gamified learning* (GL), which allows including in the educational sphere social activities that have the capacity to entertain and develop knowledge in students who use it (Bainbridge et al., 2022). Carried out in an online environment, this work connects with the contribution of Urh et al. (2015), who established that gamification enables adjusting e-learning teaching to the personal needs of the learner. For these authors, the game is a system based on rules capable of specifying the way players interact with the game world itself, which is articulated through mechanics such as points, levels, missions or leaderboards and their relationships, as well as dynamics such as competition, collaboration, community or collection (Urh et al., 2015). It is important to note that game-based learning and serious games differ from gamified learning. While gamified learning tends to use game-like mechanics or components, including scores applied to real environments (Ghai & Tandon, 2022), game-based learning and serious games consist of full featured games, the main purpose of which is training, practice and interactions (Karagiorgas & Niemann, 2017; Krath et al., 2021). This methodology is included in the process to enhance interactions and facilitate contact between students in a relaxed manner and more laid-back environment.

The third educational methodology is *online learning* (OL), also called online education. Online learning and online education practices have been widely defined in the last three decades, but in most cases without clearly distinguishing them (Singh & Thurman, 2019). Both concepts are still grappling in parallel with the growth of technology. However, the essential elements for defining online learning are the use of technology, the articulation of synchronous or asynchronous environments, interactivity/learning activities, and the role of physical distance, while online education is mostly used to describe a non-physical framework for teaching (Singh & Thurman, 2019). Some research studies have confirmed that these online learning resources can increase student participation and satisfaction in both educational activities and their social interactions (Çebi, 2022). Other studies show how online learning resources help improve students’ academic performance (Paulsen & McCormick, 2020) and emotional and cognitive commitment (Vahedi et al., 2021). In our study, this third methodology was imposed by government regulations due to the period of confinement we were going through.

**Fig. 1:**
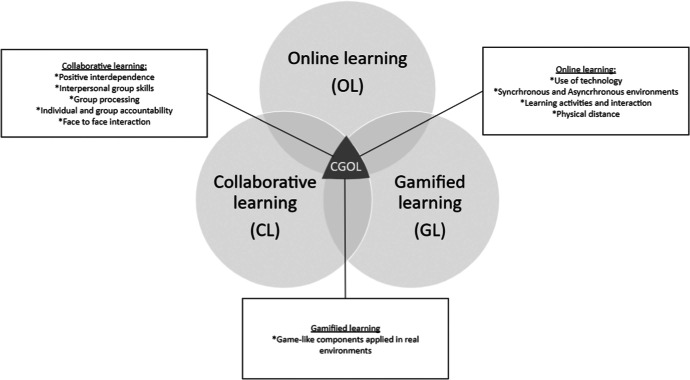
Collaborative and Gamified Online Learning (CGOL) elements

To summarise, the teaching method applied in this study is a *Collaborative and Gamified Online Learning* (CGOL) methodology, that is the result of combining the three above mentioned methodologies (CL + GL + OL: CGOL). Figure 1 is a simple preliminary computer graphic that provides a broad overview of all the characteristics it includes. A CGOL methodology only exists in the intersection between the three methodologies mentioned and excludes the use of only two of the three or partial combinations between them.

In this paper, we address the possible effects of CGOL on emotional well-being in higher-education students. Previous and recent reviews of the specific literature show mixed evidence, even though they tend to highlight the positive impacts on health and well-being (Johnson et al., 2016) and the role of CGOL as a facilitator of students’ emotional and cognitive learning (Torres-Toukoumidis et al., 2023). With the aim of analysing in greater depth the emotional and social aspects, this study’s proposed model is founded on an extension of Social Learning Theory based on Control-Value Theory of Achievement Emotions.

The control-value theory of achievement emotions (Pekrun, 2006) assumes that students’ emotions play a fundamental role in their learning, upholding the idea that the establishment of a positive atmosphere amongst students and the creation of emotional bonds between teachers and students provokes an increase in student performance and greater satisfaction and enjoyment with their own learning (Berweger et al., 2022). In this theory, *‘Control’* refers to a student’s perception and judgement of his/her capacity to affect the learning process and the results, whereas *‘Value’* refers to the relevance a student attributes to the learning task or results (Wu et al., 2021). In addition, said theory postulates that those methods can increase control of the activity in terms of competence and self-efficacy, and of engagement and involvement, enabling deeper knowledge of what has been learned (Darling-Hammond et al., 2020; Saqr et al., 2020).

On the other hand, the components of the social learning theory are attitudes, values and orientations that conform individual behaviours (Akers & Jennings, 2015). According to the social learning perspective, knowledge is not only formed through an individual’s attitude, but also while individuals interact and collaborate, highlighting the role of these interactions as a key aspect for a successful learning experience (Henning, 2004; Hill et al., 2009). Although this theory focuses on students’ social interactions instead of specific valuable experiences or emotional constructs and their relationships, it has been applied in environments and situations other than the ones that exist in the traditional classroom. This is the case of collaborative and gamified learning methodologies, which when used in cultural marketing (Ha et al., 2021) or education (Dikcius et al., 2021), have demonstrated their capacity to reinforce collaborative group interactions and the satisfaction and commitment of student learning.

To further understand how social interactions can facilitate positive experiences and emotional attractiveness, it is necessary to incorporate the control-value theory emotional measuring constructs to the social learning conceptual framework. In applying the control-value theory of achievement emotions in our study, we distinguish between student ‘control’, that is, his/her decision to participate (or not) in the different CGOL activities proposed during the semester to foment social interactions and generate a positive atmosphere; and ‘value’ (academic achievement or learning performance), that is, class- and test-related emotions.

The positive effects of students´ attitude and their social interaction on learning have been widely confirmed when using computer tool-based learning (see e.g., Kreijns et al., 2003; Reed et al., 2010). Nevertheless, even though there is a growing interest in the learning literature focused on the links between students’ attitude and emotions (Mirahmadizadeh et al., 2020), little is known about the effects of students’ attitude and social interactions on class- and test-related emotions.

### Attitude as a second order construct

In the field of e-learning, there is evidence which suggests that students’ *attitude*, construed as ‘the evaluative students’ reactions, favourable or unfavourable, towards engaging in the target behaviour’ (Hagger & Chatzisarantis, 2005), might impact not only on the adoption of information technology, but also on academic performance (Aguilera-Hermida, 2020). Specifically, for the relationship between attitude and emotions, previous literature highlights that those educational programs are essential for encouraging an optimistic attitude and accelerating positive emotions during an individual’s learning process (Mirahmadizadeh et al., 2020; Yang et al., 2022). In this line, Mirahmadizadeh et al. (2020) also suggest that practitioners should develop strategic plans with the specific purpose of finding the weak points of educational systems, especially in pupils with stronger negative emotions. These plans are also considered an appropriate method for improving students’ learning performance during a global health crisis with periods of social distancing.

Attitude is a commonly studied construct in educational research and is often used as a predictor of behavior (Tatnall & Fluck, 2022). In some cases, attitude is treated as a second order construct, which means that it is seen as a higher-level construct that is made up of several sub-components or dimensions. For example, in the Technology Acceptance Model, attitude is seen as a second order construct made up of two sub-components: perceived usefulness and perceived ease of use (Davis, 1989). These two sub-components combine to form the overall attitude towards technology. Furthermore, Hagger and Chatzisarantis (2005), on measuring attitude as a (second)-order reflective-formative construct operationalised with instrumental and affective attitude, reported that the second-order models exhibited the most optimal parsimony-corrected fit indices. They also offer a framework around attitude based on two core concepts: ‘evaluative students’ favourable or unfavourable reactions’, and ‘engaging in the target behaviour’. In this study, attitude is also proposed as a higher (second)-order reflective-formative construct with three lower (first)-order reflectively measured constructs: *perceived usefulness, entertainment and habit*.

On this basis, attitude requires that students perceive that the knowledge to be obtained is (i) significant and useful (*perceived usefulness*). In order to achieve this, different studies show the need to previously increase students’ emotional engagement and motivation by means of techniques that foment empathy and self-reflection (Tan et al., 2022), and the capacity to resolve and channel the possible conflicts that could arise amongst students (Schnaubert & Bodemer, 2019). In the specific literature, Boateng et al. (2016) used data collected from a questionnaire administered to 337 students at the University of Ghana to support the idea that perceived usefulness has a direct relation with attitude in respect to the use of new technologies. The same conclusion was reached by Muñoz-Carril et al. (2021), who on the basis of the Partial Least Squares (PLS) method indicated that perceived usefulness in collaborative methodologies affected students’ attitude and their learning. Lastly, Lee et al. (2021) reviewed the literature to analyse the reasons that justify the acceptance of online learning amongst students, highlighting that one of these reasons is the perceived usefulness of this type of learning.

Furthermore, the capacity of education to affect students’ beliefs and attitudes through (ii) *entertainment* making use of traditional criteria, formulations and adapted logic, has also been analysed in the literature. Slater and Rouner (2002), who extend the vision of Bandura’s social cognitive theory of 1986, believe that entertainment-education to influence social values and attitudes are justifiable and established a relationship between entertainment and education. They highlight the capacity of education with entertainment to affect students’ beliefs and attitudes. In addition to that study, others that are not directly connected to the application of educational methods indicate a direct relationship between entertainment and attitude (Waiguny et al., 2012).

Finally, (iii) *habit* results from the relation between behaviour in a situation of learning acquired through practice, and its repetition. Habit affects the entire learning process, in addition to being connected to students’ personal characteristics, feelings and surroundings (Schmidt & Čreslovnik, 2010). Concerning the postulated relationship between habit and attitude, previous literature confirms that habits are functional, supported by actions in the past that have positive consequences; thus, many habits may be associated with positive attitudes (Verplanken & Aarts, 1999). Previous research confirms the frequency of the effect of past behaviour on future performance and postulates that it may contribute to intentions and behaviours guided by intentions (Ouellette & Wood, 1998). In respect to the learning atmosphere, research such as the one carried out by Greene et al. (2021) is considered. Their study focused on the efforts made by North American university students to obtain adequate online learning, highlighting the need for this habit to increase the possibility of acceptance of these procedures. Furthermore, after administering a questionnaire to students of Fine Arts in Singapore, Koh and Kan (2021) considered that students’ habits had a strong influence on learning with technological resources.

### Social interaction

The other predictor of students´ participation in CGOL used in this study is their social interaction. Due to the predominant cognitive perspective on pedagogical methodologies for the last 50 years (Hill et al., 2009), one main pitfall of collaboration and social interaction is the assumption that social interaction is only a form of learning in cognitive processes, misjudging the effects of collaboration as a way to learn from a valuable experience (Kreijns et al., 2003). Social interactions generate the feeling that each member’s individual actions affect the group and (Alonso et al., 2019) and facilitate students’ critical and reflexive learning in an individual and collective manner. However, only defining working groups is not a guarantee for collaboration, and some incentives need to be defined within the groups (Kreijns et al., 2003). Many authors also postulate that social interaction is an important factor in remote group learning, suggesting that it is the key for collaboration (Liaw & Huang, 2000; Northrup, 2001; Kreijns et al., 2003). Furthermore, the specific literature confirms that the methods that seek to foment social interaction achieve deeper and longer-lasting learning attitudes through students’ autonomy and by enhancing the creativity of tasks (Passyn & Billups, 2019).

### Research purpose and formation of hypotheses

The research purpose of our study is to analyse whether the use of CGOL methodologies contributes to reduce first-year academic emotional problems. To this end, we first try to check if the students´ attitude—comprising perceived usefulness, entertainment and habit-—and the social interaction these methodologies promote are related to students’ intention to participate in CGOL activities. Moreover, we analyse if this active participation in CGOL contributes to reduce students’ academic emotions in class and on tests.

The existing literature offers a strong conceptual background on the relationships proposed in this study’s research model. In order to determine the validity of this combination of educational elements, the following hypotheses have been established.

**Fig. 2:**
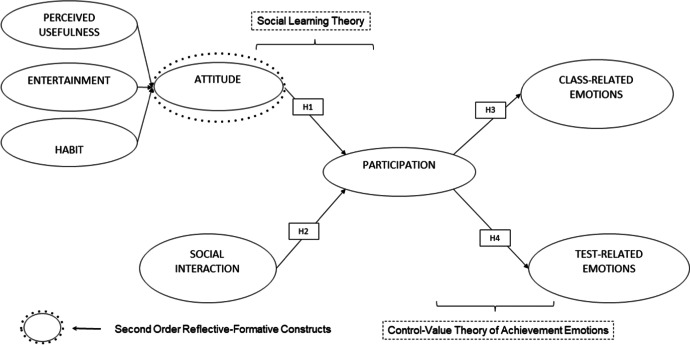
Proposed model of CGOL participation of integrated concepts of Social Learning Theory and Control-Value Theory of Achievement Emotions

In this model, students’ attitude in the use of the online methodology is operationalised through perceived usefulness, entertainment, and habit. By operationalising attitude as a higher (second)-order reflective-formative construct, this study offers a unique perspective of attitude made up of these three lower (first)-order reflectively measured constructs. Higher order constructs reduce collinearity issues and help in the interpretation of results (Hair et al., 2017). It also reduces the number of hypothesised relationships and generates reliable and valid empirical results (Thien, 2020). All three dimensions, each with a different set of items, have different conceptual meanings as reflected in their measures and are thus measured as reflective first order constructs. Following Jarvis et al.’s (2003) guidelines, and according to the direction of causality, interchangeability of the items and their covariation, it can be deduced that attitude is a higher (second)-order formative construct that is measured by three reflective lower (first) order constructs depicted in Fig. 2. Finally, following the nature and objectives of this study, combining these three independent variables reduces the number of paths and improves the readiness and interpretation of the results. Furthermore, in the previous literature, attitude is extensively related with participation. In the scientific literature on education, this idea is linked to the one reflected in Darling-Hammond et al. (2020). Bearing in mind the implications of the emerging, cross-disciplinary body of knowledge called Science of Learning and Development for student learning, they point out that attitudes link academic efforts with students’ personal values. Additionally, Aguilera-Hermida et al. (2021) analysed the factors that determined online learning amongst students based on data provided in 109 questionnaires answered by students in four countries, concluding that said students’ attitude towards e-learning was considered relevant for their cognitive engagement. Therefore, the following hypothesis is formulated:

- H1: The better the attitude is, the greater students’ participation in CGOL is.

Hypothesis 2 (H2) considers the influence of social interactions on students’ participation. In respect to the educational sphere, previous studies such as Cerratto Pargman et al. (2018) highlight students’ capacity to configure technological resources as collaborative instruments by establishing multiple instrumental mediations between teachers and students. Furthermore, the work carried out by Strauß and Rummel (2021) with data obtained from German university students supports this interpretation, incorporating the need for prior support, regulation that is appropriate for the collaboration, and constant interaction to increase the satisfaction of interactions with digital resources. These conclusions support this hypothesis’ aim to determine whether the existence of social interactions of students with teachers, amongst others, predicts the use of proposed online educational resources and participation in CGOL. Therefore, in accordance with the previous literature, the following hypothesis is formulated:

- H2: The better the social interaction is, the greater students’ participation in CGOL is.

Finally, hypotheses H3 and H4 study the relationship of students´ participation in CGOL with class- and test-related emotions, respectively. Hypothesis 3 (H3) aims to verify whether participation in the proposed CGOL has a positive effect on class-related emotions. Several studies have focused on this issue, such as Garnett et al. (2017), using control-value theory of achievement emotions to obtain predictors of physical education students’ behaviour and emotional engagement in their classes. Specifically, they studied changes in students’ participation in classes during one semester. Their results showed that students’ engagement was a fundamental factor to generate positive and internalised attitudes of the positive effect of attending classes. In this line, additional practice and better outcomes have been found in previous literature (Bergdahl & Bond, 2022). Thus, in accordance with control-value theory and previous research, the following hypothesis is formulated:

- H3: The greater students’ participation in CGOL is, the better students’ class-related emotions are.

Lastly, Hypothesis 4 (H4) analyses whether participation in the proposed CGOL has a positive effect on test-related emotions. In this sense, Boulton et al. (2018) determined, with data obtained from undergraduate students at the University of Exeter, that a high degree of activity in a virtual learning setting is associated with higher marks, although a low degree of activity does not necessarily entail lower marks. More recently, Venkatesh et al. (2020) used data provided by first-year university students to conclude that students’ academic performance improved thanks to a combination of collaborative methods and the use of remote learning through technological resources, indicating as well that the performance expectations affected their satisfaction with regard to these combined methods. Thus, in accordance with the control-value theory and previous research, the following hypothesis is formulated:

- H4: The greater students’ participation in CGOL is, the better students’ test-related emotions are.

Figure 2 shows the research model designed. This model aims to generate knowledge about the influence of individual attitude and social interactions on participation in CGOL and about the influence of participation in designed CGOL activities on students’ class- and test-related emotions.

## Methodology

The data were analysed using the PLS technique and SmartPLS 4.0.8.3 software (Ringle et al., 2015). The use of this technique is recommended when the structural model is complex and includes many indicators and model relationships (Hair et al., 2019). Furthermore, with this technique we estimate the sign and significance of the different hypothesised relationships between the constructs of the structural model and evaluate the reliability and validity of the measurement model (Barroso et al., 2010), which is the primary objective of this study. Another reason for selecting Partial Least Squares-Structural Equation Modelling (PLS-SEM) is that it can handle non normal data (Hair et al., 2019). Our analysis of multivariate normality (Mardia, 1970) showed that Mardia’s multivariate skewness (*β* = 8128.7; *p* < 0.001) and multivariate kurtosis (*β* = 86.7669; *p* < 0.001) suggest the presence of multivariate non-normality.

### Participants

This study uses quantitative research for surveying first-year university students. The sample units are students at a Spanish University enrolled in two degree programmes of the Schools of Economics and Law that shared a common principle: online sessions in which CGOL activities were applied across all the units.

Specifically, the CGOL activities were geared at first-year students enrolled in the same subjects taught during both the first and second semesters. Each CGOL methodology was adapted to the units and main concepts addressed in each subject. All the students included in the research were taught during the academic year 19–20 (second semester) or 20–21 (first and second semesters) and participated in the CGOL methodology during COVID-19 lockdowns.

The study population of first-year students enrolled at the School of Economics and Business Sciences and the School of Law in the 19–20 and 20–21 academic years consisted of 3387 students. The proportion of students enrolled by degree is 53.9% of students at the School of Economic and Business Sciences and 46.1% of students at the School of Law. The sample size consisted of 450 individuals, and following the sample collection process, 171 valid responses were obtained from students of Economics and Business Sciences and 130 valid responses were collected from Law students. To clarify the sample’s descriptive characteristics, since some universities are specialised in specific population segments such as middle age or professionals, detailed information of the students involved in the study is shown in Table 1. With a final sample size of 301 individuals, a sampling error of 5.39% is assumed with a confidence interval level of 95%.

**Table 1:** Descriptive results

Variables	%
Academic year	
19–20	28.6%
20–21	71.4%
School	
Law	56.1%
Economics and Business Sciences	43.9%
Age	
25 or less	92.7%
From 26 to 35	4.7%
From 36 to 45	2.0%
From 46 to 55	0.6%
56 or older	0.0%
Sex	
Male	39.9%
Female	60.1%
Marital status	
Married	1.3%
Divorced	1.0%
Common-law couple	4.0%
Single	93.7%
Occupation	
No occupation	0.0%
Occupied (studying)	98.0%
Unemployed	2.0%

### Settings and educational process

Each CGOL activity designed was presented to the students after working on the related unit content. At least 2 hours of each unit were used for the activity, and when needed, teams could finish the activity working after class time. The process of delivery was as follows: after finishing the theoretical explanations, the teachers explained the section or unit activity and formed groups randomly to work online and separately in group online mode according to CGOL methodology. The instructions and materials for each game were previously uploaded to the platform. A forum was opened in each unit for uploading team results. Students had to upload their outcomes before the end of the lesson’s t time or during that afternoon. At the end of the day and before beginning the next activity, the teachers assessed the activities with 1 to 10 points according to their accuracy. Tables 2 and 3 show games designed and their connection to course content, and the gamification and collaborative elements included, respectively.

**Table 2:** CGOL Activities

Concepts and types of activities	Explanation of activities
School of Business and Management	
Section 1. Introduction Unit 1: Needs, desires and wants Unit 2: Analysis of environment	U1. Using an online dice, players play on a given board. To win their game, they have to give correct examples of the application of concepts, according to the box theme. U2. Using an online dice, players play on a given board. To win their game, they have to give correct examples of SWOT elements, according to the box theme.
Section 2. Business Information Systems and Market research Unit 3: Business Information Systems Unit 4: Market research	U3. Using an online dice, players play on a given board. To win their game, they have to give correct examples to adapt their business information systems to an online environment, according to the box theme. U4. Using an online dice, players play on a given board. To win their game, they have to give correct examples of elements from a questionnaire for market research purposes, according to the box theme.
Section 3. Consumer behaviour and market segmentation Unit 5: Consumer behaviour Unit 6: Market segmentation	U5. Using an online dice, players play on a given board. To win their game, they have to give correct examples of perception elements for advertisements, according to the box theme. U6. Using a card-shuffler randomly, players work with 3 cards, where each card’s characteristic represents a different market variable (age, sex, personality). To win their game, they have to give correct examples of a segmentation strategy and define a Marketing Mix, according to the box theme.
Section 4: Marketing plan Unit 7: Action plan	U7. Using an online dice, players play on a given board. To win their game, they have to give correct examples of action plan elements, according to the box theme.
School of Law	
Section 1: Introduction to Economics	Using an online dice, players play on a given board. They have to solve the designed quiz and explain basic economic concepts presented during the theoretical classes through mimicry (such as ‘profit motive’, or ‘oligopoly’, based on the game ‘Guesstures’) and drawings (such as ‘equilibrium price’, or ‘Production Possibilities Frontier (PPF)’, based on the game ‘Pictionary’). To win their game, they have to give correct examples of economic concepts, according to the box theme.
Section 2: Microeconomics	Using an online dice, players play on a given board. They have to solve the designed quiz and define microeconomic concepts (e.g., ‘price elasticity’ or ‘marginal costs’) without using certain words (based on the game ‘Taboo’). To win their game, they have to give correct examples of microeconomic concepts, according to the box theme.
Section 3: Macroeconomics	Using an online dice, players play on a given board. They get an Escape Room consisting of solving a set of macroeconomic problems based on fiscal and monetary policies. To win their game, they have to give correct examples of macroeconomic problems, according to the box theme.
Section 4: Problems of Contemporary Economies	Using an online dice, players play on a given board. They have to solve the designed quiz by calculating its Human Development Index with the tools of the United Nations Development Programme (UNDP). To win their game, they have to calculate indexes according to the box theme.

**Table 3:** Elements, mechanics and dynamics of gamification in CGOL methodologies and activities

Game elements:	1. Rule-based quiz and games 2. Clear and meaningful goals for each activity 3. Voluntary participation	Each game’s rules and goals were defined in advance and shared with students before participating. As the game started after the theoretical section of the lesson, students not interested in participating could leave the online assignment.
Game mechanics:	1. Leaderboards	Games are based on 20 boxes of leaderboards. A correct answer makes you move forward, and an incorrect answer doesn’t.
Game dynamics included:	1. Competition	The first player to finish each game wins.
Collaborative elements:	1. Face to face interaction 2. Group processing	Face to face interaction is needed in each quiz/game. The final results of each match (final quiz solution given and scores) are uploaded to the platform subject forum when the game is finished for subsequent classmate processing.

After having completed all those procedures and taken an exam at the end of the term, the students answered a questionnaire to evaluate the whole CGOL methodology designed, and their class- and test-related emotions. During those two years, and due to COVID-19 limitations, the weight of continuous assessment changed and was higher than in previous years, becoming the sole assessment methodology for those students participating in this evaluation system (others could directly take the final exam to obtain their grade in the subject). As a result, students could only pass the subject if they had more than 5 out of 10 points in both the CGOL activities and the final exam. The CGOL activities entailed 40% of the final grade and the exam entailed 60%. In addition, for the first time the preparation of the subject was entirely online. The two classes followed this CGOL and continuous assessment methodology for three semesters. Due to these circumstances, we try to assess whether the emotional response to the class and tests could be positive based on the whole CGOL methodology designed, instead of focusing on the heterogeneity of each specific CGOL activity.

### Measurement instrument

The questionnaire was developed to cover the purpose of the study, based on previous literature. To validate the questionnaire, 10 experts on the subject evaluated the content validity, and a pre-test was launched with a small group of 20 students. As a result of the validation process, 2 questions and 4 ambiguous concepts were modified.

The final questionnaire for the collection of information is composed of 7 constructs: Perceived Usefulness (PU), Entertainment (ENT), Habit (H), Social Interaction (SI), Participation (P), Class-Related Emotions (CRE) and Test-Related Emotions (TRE). As shown in Table 4, all constructs were measured with three or more variables. The measurement variables were adapted to the context of online learning to be included in the questionnaire, and Pekrun’s Achievement Emotions Questionnaire was followed to develop the two constructs related to emotions (CRE and TRE). We decided to only study class- and test-related emotions, since the last construct of the Achievement Emotions Questionnaire, learning emotions, is mostly related with cognitive and affective drivers, and the changes in our methodology were primarily related with the place where the subject was taught and the tests were delivered, going from a physical environment to an online environment.

**Table 4:** Variables and Dimensions

Variables and Dimensions	Number of items and categories	Authors
Sociodemographic	Age	Adapted from Vallespín et al. (2017)
	Categories: 18–25; 26–35; 36–45; 46–55; 56–65, over 65	
	Sex	
	Categories: male – female	
	Level of completed studies	
	Categories: no studies; primary school studies; secondary school studies; undergraduate studies; postgraduate studies	
	Marital status	
	Categories: married; single; widow/widower; divorced; common-law partnership	
	Occupation	
	Categories: employed; unemployed; retired, university student; disabled; housewife	
Perceived Usefulness	V1.2 Please indicate the extent to which you agree with each of the following statements (1 strongly disagree - 7 strongly agree):	Adapted from Liaw and Huang (2013)
	V.1.2.1 These CGOL have great value.	
	V.1.2.2 These CGOL have been very useful for my learning.	
	V.1.2.3 These CGOL are very inspiring to learn.	
	V.1.2.4 These CGOL are perfect for keeping an overview of the learning process.	
Entertainment	V1.1 Please indicate the extent to which you agree with each of the following statements (1 strongly disagree - 7 strongly agree):	Adapted from He et al. (2018)
	V.1.1.1 Working with these CGOL is very entertaining.	
	V.1.1.2 Working with these CGOL is catching, it picks me up.	
	V.1.1.3 Working with these CGOL is not just a way of learning, it entertains me.	
Habit	V1.3 Please indicate the extent to which you agree with each of the following statements (1 strongly disagree - 7 strongly agree):	Adapted from Gefen (2003)
	V.1.3.1 These are the CGOL I usually use instead of others.	
	V.1.3.2 These are my preferred CGOL instead of others.	
	V.1.3.3 When I need to work with the content of a unit, these are the CGOL I use first.	
	V.1.3.4 I often use CGOL resources for learning.	
Social Interaction	V1.4 Please indicate the extent to which you agree with each of the following statements (1 strongly disagree - 7 strongly agree):	Adapted from Tu et al. (2019)
	V.1.4.1 These CGOL allow me to interact with my classmates.	
	V.1.4.2 These CGOL allow me to do activities with my classmates.	
	V.1.4.3 The teacher can evaluate the group work through these CGOL.	
Participation	V.1.5 Please indicate the extent to which you agree with each of the following statements (1 strongly disagree - 7 strongly agree):	Adapted from Rese et al. (2017)
	V.1.5.1 After using these CGOL activities, I now know I would use these CGOL activities immediately when available.	
	V.1.5.2 After using these CGOL activities, I know now I would give priority to these CGOL activities over other resources.	
	V.1.5.3 After using these CGOL activities, I now know that if I were to learn online in the future, I would give priority to methodologies including these CGOL activities over other methodologies.	
	V.1.5.4 After using these CGOL activities, I now know that I would recommend using these CGOL activities to my classmates.	
	V.1.5.5 After using these CGOL activities, I now know that I would use these CGOL activities regularly in the future.	
Class-related emotions*	V.2.1.1a I enjoy being in class. (d)	Achievement Emotions Questionnaire. Adapted from Pekrun et al. (2011)
	V.2.1.1b I am confident when I go to class. (b)	
	V.2.1.1c I am proud of myself. (a)	
	V.2.1.1d I am angry. (a)	
	V.2.1.1e Thinking about class makes me feel uneasy. (b)	
	V.2.1.1f I get embarrassed. (d)	
	V.2.1.1 g I feel hopeless. (b)	
	V.2.1.1 h I get bored. (d)	
Test-related emotions*	V.2.1.2a For me the test is a challenge that is enjoyable. (d)	Achievement Emotions Questionnaire. Adapted from Pekrun et al. (2011)
	V.2.1.2b I have great hope that my abilities will be sufficient. (b)	
	V.2.1.2c I’m proud of how well I mastered the exam. (a)	
	V.2.1.2d I feel very relieved. (a)	
	V.2.1.2e I am fairly annoyed. (a)	
	V.2.1.2f I feel panicky when taking an exam. (d)	
	V.2.1.2 g I feel ashamed. (a)	
	V.2.1.2 h I have lost all hope that I have the ability to do well on the exam. (d)	

Source: own development based on the literature

*(a) after; (b) before; d (during)

Six sociodemographic variables were used to collect information about the respondents, namely age, education level, gender, household income, marital status and occupation. We designed different formats according to the variable that was being measured; for example, to measure gender, a nominal variable was used. However, to collect the responses of the constructs included in the model, 7-point Likert scales were used in all cases, ranging from (1) totally disagree to (7) totally agree.

### Data collection

The information was collected through the self-administered questionnaire handed out during classes by the researchers participating in this study at the end of the academic period. Based on the researchers’ proximity to the students, a non-probabilistic sampling method was employed (convenience) according to the selection criteria of the sample elements. This technique was considered to be the most appropriate data collection method for the study.

### Measurement model estimation

#### Common method bias

Common method bias is typically associated with data derived from a single source (Avolio et al., 1991) and can pose issues in self-reported quantitative studies (Spector., 2006). Common method bias can have a negative impact on validity (MacKenzie & Podsakoff, 2012) and can affect structural relationships (Kline, 2016). Statistical control (Reio, 2010) is an approach to minimise the risk of common method bias. With the aim of checking the Common Method Bias, the study employs Harman’s single factor test. The total variance explained by single items is less than 50% and according to Harman’s test confirms the absence of a common method bias (Fuller et al., 2016; Podsakoff et al., 2012).

#### Assessment of reflective constructs

Table 5 shows the values of each measurement model item in the first-order reflective constructs. There is high internal consistency, as demonstrated by a composite reliability of >0.7 and Cronbach alpha of >0.7. Convergent validity is established with average variance extracted (AVE) >0.5 (Hair et al., 2017). Additionally, regarding the primary components, all of the primary components of the first-order reflective construct exceed the minimum threshold value, greater than or equal to 0.707 (Hair et al., 2011), except item 1.3.4 in Habit and 2.1.1f in Class-Related Emotions (with values lower than 0.6 in both cases), so the scales were refined by eliminating both items.

**Table 5:** Variables, dimensions, loads, composite reliability and average variance extracted (AVE)

	Dimension	Loading	Cronbach’s Alpha	CR(ρ_c)_	AVE
Entertainment (ENT)	1.1.1	0.952	0.927	0.954	0.873
	1.1.2	0.937			
	1.1.3	0.914			
Perceived Usefulness (PU)	1.2.1	0.907	0.931	0.951	0.828
	1.2.2	0.919			
	1.2.3	0.901			
	1.2.4	0.913			
Habit (H)	1.3.1	0.926	0.9	0.937	0.833
	1.3.2	0.905			
	1.3.3	0.907			
Social Interaction (SI)	1.4.1	0.871	0.849	0.909	0.769
	1.4.2	0.933			
	1.4.3	0.824			
Participation (P)	1.5.1	0.926	0.96	0.969	0.863
	1.5.2	0.906			
	1.5.3	0.942			
	1.5.4	0.941			
	1.5.5	0.93			
Class-related emotions (CRE)	2.1.1a	0.88	0.923	0.938	0.684
	2.1.1b	0.841			
	2.1.1c	0.834			
	2.1.1d	0.828			
	2.1.1e	0.771			
	2.1.1 g	0.822			
	2.1.1 h	0.811			
Test-related emotions (TRE)	2.1.2a	0.742	0.914	0.93	0.624
	2.1.2b	0.762			
	2.1.2c	0.868			
	2.1.2d	0.744			
	2.1.2e	0.844			
	2.1.2f	0.753			
	2.1.2g	0.768			
	2.2.1h	0.828			

The reliability of the constructs was evaluated through composite reliability (ρc), whose values must be greater than 0.7. As these values are higher in all the model constructs, a high internal consistency is confirmed (see Table 5). Finally, convergent and discriminant validity were evaluated. An AVE value greater than 0.50 establishes that more than 50% of the variance of a construct is explained by its indicators (Hair et al., 2011, 2014). The mean extracted variances of our constructs exceed the value 0.5 in all cases.

To analyse the discriminant validity, three procedures were used: the Fornell-Larcker criterion, the criterion of the cross loads and the Heterotrait Monotrait Ratio criterion. The Fronell-Larcker criterion confirms that the AVE of each latent construct is greater than the variance that said construct shares with the model’s other constructs (Hair et al., 2011). Thus, the correlations between the constructs are found to be lower than the square root of the AVE (See Table 6).

**Table 6:** Discriminant validity of first-order constructs –Fornell-Larcker’s criterion

	CRE	ENT	H	SI	P	PU	TRE
CRE	**.827**						
ENT	.602	**.934**					
H	.539	.755	**.913**				
SI	.426	.497	.522	**.877**			
P	.549	.740	.768	.542	**.929**		
PU	.609	.741	.696	.542	.710	**.910**	
TRE	.508	.491	.448	.414	.539	.446	**.790**

Note: The off-diagonal values (bold) in the above matrix are the square correlations between the latent constructs

The second criterion examines if each indicator loads more heavily on its construct than on the rest of the model’s latent variables (Hair et al., 2011). As shown in Table 7, this criterion is validated. Table 8 shows the third criterion (Heterotrait Monotrait Ratio), where all correlations between constructs are less than 1.00. Therefore, according to Richter et al. (2016), there exists discriminant validity.

**Table 7:** Cross loadings Matrix

Dimension	Entertainment	Perceived Usefulness	Habit	Social Interaction	Participation	Class-related Emotions	Test-related emotions
1.1.1	**0.952**	0.688	0.700	0.463	0.690	0.560	0.445
1.1.2	**0.937**	0.694	0.727	0.469	0.702	0.564	0.483
1.1.3	**0.914**	0.695	0.688	0.461	0.682	0.564	0.447
1.2.1	0.657	**0.907**	0.625	0.508	0.635	0.567	0.369
1.2.2	0.672	**0.919**	0.673	0.504	0.652	0.575	0.424
1.2.3	0.696	**0.901**	0.619	0.478	0.651	0.526	0.420
1.2.4	0.672	**0.913**	0.616	0.482	0.646	0.549	0.408
1.3.1	0.679	0.654	**0.926**	0.507	0.720	0.507	0.424
1.3.2	0.732	0.635	**0.905**	0.462	0.693	0.479	0.397
1.3.3	0.654	0.615	**0.907**	0.459	0.690	0.490	0.408
1.4.1	0.491	0.516	0.446	**0.871**	0.512	0.380	0.391
1.4.2	0.457	0.499	0.484	**0.933**	0.481	0.343	0.339
1.4.3	0.349	0.400	0.444	**0.824**	0.426	0.403	0.359
1.5.1	0.689	0.680	0.724	0.495	**0.926**	0.505	0.502
1.5.2	0.685	0.619	0.724	0.485	**0.906**	0.473	0.495
1.5.3	0.667	0.645	0.705	0.498	**0.942**	0.529	0.515
1.5.4	0.703	0.678	0.712	0.528	**0.941**	0.532	0.506
1.5.5	0.694	0.676	0.705	0.510	**0.930**	0.511	0.486
2.1.1a	0.514	0.528	0.462	0.332	0.494	**0.880**	0.401
2.1.1b	0.503	0.512	0.470	0.432	0.482	**0.841**	0.440
2.1.1c	0.505	0.501	0.433	0.368	0.461	**0.834**	0.479
2.1.1d	0.535	0.489	0.476	0.382	0.466	**0.828**	0.432
2.1.1e	0.449	0.427	0.397	0.307	0.401	**0.771**	0.368
2.1.1g	0.507	0.558	0.475	0.337	0.451	**0.822**	0.465
2.1.1h	0.468	0.507	0.402	0.301	0.415	**0.811**	0.350
2.1.2a	0.515	0.442	0.430	0.346	0.503	0.482	**0.742**
2.1.2b	0.437	0.410	0.421	0.361	0.458	0.460	**0.762**
2.1.2c	0.396	0.306	0.324	0.313	0.406	0.380	**0.868**
2.1.2d	0.327	0.293	0.348	0.291	0.404	0.394	**0.744**
2.1.2e	0.410	0.324	0.339	0.365	0.439	0.382	**0.844**
2.1.2f	0.305	0.299	0.273	0.254	0.363	0.336	**0.753**
2.1.2g	0.307	0.339	0.319	0.305	0.351	0.317	**0.768**
2.1.2h	0.340	0.363	0.337	0.352	0.433	0.409	**0.828**

Note: Factor loadings in bold

**Table 8:** Discriminant validity of first order constructs (HTMT)

	CRE	ENT	H	SI	P	PU
CRE						
ENT	.650					
H	.590	.825				
SI	.482	.555	.597			
P	.581	.784	.827	.597		
PU	.657	.798	.760	.605	.751	
TRE	.544	.523	.488	.465	.568	.476

#### Assessment of formative constructs

In this research study, attitude was introduced as a higher-order construct of type two (reflective-formative). To evaluate this construct, its aggregate values were used (Wright et al., 2012). At this point of the model validation process, the measurement model had to be re-evaluated to test the nomological structure. As Table 9 shows, the composite reliability (ρc) presented a value over 0.70. All items’ λ, in turn, are higher than 0.60 (Hair et al., 2014). Finally, convergent validity was assessed via the items´ AVE, showing that all values were over 0.50 (Hair et al., 2011, 2014). Moreover, all the variance inflation factor (VIF) values of the predictor constructs, Perceived Usefulness, Entertainment, and Habit, were below 3, showing that collinearity is not an issue between them (Hair et al., 2019).

**Table 9:** Analysis of second-order model’s individual reliability, composite reliability and convergent validity

	Loading	Cronbach’s Alpha	CR(ρ_c)_	AVE
AT		0.95	0.957	0.692
ENT	0.912			
PU	0.914			
H	0.889			

#### Assessment of the structural model

To evaluate the results of the structural model, we evaluated the significance of the path coefficient, the R-square, and the predictive relevance, Q-square.

Validation of the structural model starts with the examination of R^2^ values and the Stone-Geisser test (Q^2^), both reported in Table 10. The R^2^ value indicates the amount of variation of the construct explained by the model, reaching values greater than 0.1, which are considered valid (Falk & Miller, 1992). Another measure of predictive power is the Q^2^ test: if the value is greater than 0, there is predictive relevance (Hair et al., 2011). Finally, the multicollinearity of independent variables was tested using the VIF. All values for VIF were below 6.139, which is under the limit considered problematic (Vittinghoff et al., 2006). Thus, multicollinearity was not an issue in this research study (Hair et al., 2012).

**Table 10:** Variance explained and the Stone-Geisser test

	R^2^	Q^2^ (=1-SSE/SSO)
CRE	0.302	0.299
TRE	0.290	0.288
P	0.670	0.677

We also checked the Goodness of Fit index. We used standardised root mean square residuals (SRMR), which is a measure of approximate fit, to test the structural Goodness of Fit model (Henseler & Sarstedt, 2013). The value of SRMR should be less than 0.10 in the estimated model to be considered a good fit in a non-conservative interpretation (Hu & Bentler, 1999). The estimated model’s SRMR value is (SRMR = 0.095), which means that it is considered a good fit under a non-conservative interpretation.

The process finalised with the application of the confidence intervals technique to confirm the above findings. Estimated path coefficients β with confidence intervals which do not include zero allow us to reject the hypothesis that β equals zero (Henseler et al., 2009). The results obtained confirmed that all hypotheses are empirically supported, as shown in Table 11.

**Table 11:** Assessment of the structural model

	*β Coefficients*	T statistics	*P* Value	2.5%	97.5%	Sup
AT ► P	.758	20.728	<0.001	.675	.814	Yes
SI ► P	.108	2.579	0.009	.030	.195	Yes
P ► CRE	.549	10.713	<0.001	.452	.647	Yes
P ► TRE	.538	11.451	<0.001	.448	.634	Yes

**Fig. 3:**
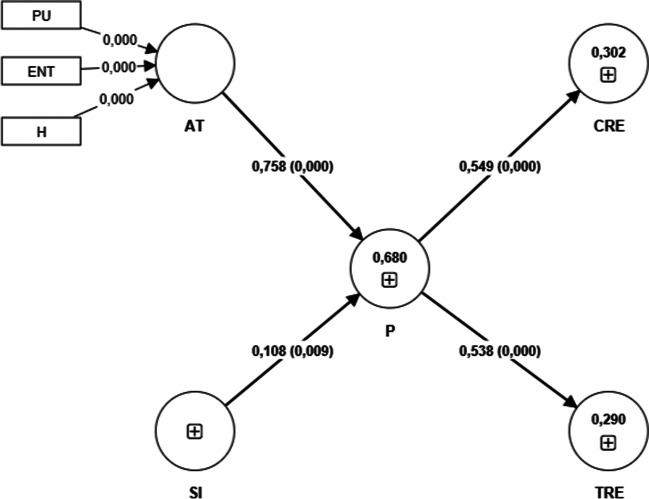
Structural model with t values

According to the results of the analysis, the proposed structural model is considered valid, and the results confirm that AT (H1) *β* = 0.758 (*t* = 20.728; *p* < 0.001) and SI, with a smaller effect, (H2) *β* = 0.118 (*t* = 2.579; *p* < 0.010) are predictors of P. In turn, P is a predictor of CRE (H3) *β* = 0.549 (*t* = 10.713; *p* < 0.001), and TRE (H4) *β* = 0.538 (*t* = 11.451; *p* < 0.001). Therefore, all direct hypothesised relationships (H1–H4) are supported (see Fig. 3).

## Discussion, conclusions, and future implications

### Discussion and conclusions

In this study we wanted to analyse whether the use of CGOL methodologies might have a positive impact on first-year university students’ emotions regarding classes and tests. To this end, using PLS-SEM modelling we analyse whether students´ attitude and social interactions contribute to enhance participation in CGOL. Secondly, we examine the association between participation in the designed CGOL methodology and students’ class- and test-related emotions.

To begin with, we find that attitude—operationalised by perceived usefulness, entertainment and habit— might be associated with greater participation in CGOL (H1), which is in line with Darling-Hammond et al. (2020) and Aguilera-Hermida et al. (2021). Thus, our results also point to the idea that perceived usefulness, entertainment and habit have an indirect and positive impact on students’ participation in CGOL resources. In consequence, educators and trainers who are facing the challenge of designing CGOL methodologies have to verify that students perceive this usefulness, which occurs specifically when these digital resources are found to be valuable, practical and to have inspired students during their learning process. Moreover, the entertainment, that is to say, the enjoyment that this digital resource brings to students is also considered an important factor when designing CGOL methodologies. Regarding habit, the third component of attitude, the results confirm the idea that students prefer to use these digital resources rather than other training alternatives, in particular when students need to prepare the content of the lessons. Furthermore, this might lead the way to finding new methodologies in which to apply some of these resources, with the aim of making university classes more enjoyable and attractive to students.

The results also show that social interactions with fellow students, that is, tasks carried out with classmates and with assessments and group evaluations by the teacher, contribute to enhance students’ participation in CGOL. The outcomes are thus in line with Cerratto Pargman et al. (2018) and Strauß and Rummel (2021), and support H2. During a period of social isolation, students probably felt that those methodologies helped them mitigate loneliness and experience ‘physical distancing’ rather than ‘social distancing’, which is in accordance with other studies carried out during the pandemic (Brambilla et al., 2021). The probable reason for a smaller effect than expected is that these first-year university students had no possibility of interacting with classmates because of confinement.

Therefore, we find that both constructs —attitude towards digital resources and social interaction— increase participation in CGOL.

We also wanted to test whether this greater participation might be associated with better student emotions regarding classes and tests. Numerous studies have evaluated and discussed the correct adaptation and performance of assessment instruments during COVID-19 (Adedoyin & Soykan, 2020; Bopegedera, 2020; Brambilla et al., 2021; Kharbat & Abu Daabes, 2021). However, the effect of adopting teaching methodologies in respect to learning and test emotions was understudied. As expected, there are also strong linkages between participation in CGOL and variables of emotions in learning and tests. From the Achievement Emotions Questionnaire developed in Pekrun et al. (2011), we find that increasing CGOL participation of students might enhance the latter’s feelings regarding classes and tests. In consequence, H3 and H4 are supported. In accordance with H3, our outcomes show that (i) before going to class participants felt confident about attending them and did not feel uneasy; while in class (ii), participants enjoyed being there and did not become embarrassed or bored; and after attending classes (iii), they were proud of themselves and did not feel uneasy. This result supports previous work such as that of Garn et al. (2017), albeit our study refers to higher education instead of childhood education. Regarding test emotions H4, we find that this greater participation might also improve test emotions and reduce anger, anxiety, and hopelessness. This result seems to be in line with Boulton et al. (2018) as well as Venkatesh et al. (2020) and provides new and complementary evidence to Roos et al. (2022).

The main conclusion of this study is that a CGOL methodology increases students’ positive academic emotions. In light of these outcomes, attitudinal and social interaction components can be considered critical elements of students’ participation in CGOL methodologies and activities, confirming them as precedents of the control element in control-value theory of achievement emotions. On the other hand, the level of participation in CGOL activities impacts students’ class and test emotions. These results provide new findings to the literature regarding computer mediated online learning and can help educators design learning scenarios to deal with student anxiety, stress and motivation.

### Theoretical recommendations

The most notable theoretical contribution of the study is the confirmation of how the attitude towards the use of collaborative and gamified learning methodologies and activities is measured for the first time in learning literature as a type two higher-order (reflective formative) construct explained by perceived usefulness, entertainment and habit in comparison with previous studies. This research thus makes a significant contribution to the learning literature by describing their interrelationships. Moreover, we also show for the first time that attitude and social interaction are precedents of participation in CGOL and confirm the positive relationship between the use of CGOL methodologies and students’ emotions before, during and after classes, and before and during the tests as well, in a context in which the students hardly know each other.

Our outcomes verify that control-value theory of achievement emotions can be used as a way to enhance social interaction theory with regard to emotions. Additionally, we include social interactions, perceived usefulness, entertainment and habit as control-related constructs for the first time. This way, we provide additional evidence for theoretical strands usually developed in the related literature (such as control-value theory or technology acceptance model), highlighting that this model is also valid during a period of social stress and compulsory isolation in which most universities were forced to implement online learning systems and teaching needs to find new paths to achieve their learning objectives.

Moreover, our study reinforces the role of academic emotions during a period of higher stress and anxiety, such as the COVID-19 pandemic, in which students face mental health challenges such as domestic problems, depression, mental issues and suicidal thoughts. Moreover, in a concrete group such as first-year university students, those problems might be especially pronounced. Using digital resources might help them reduce these possible mental health problems by enhancing their emotions towards classes and tests and improve their general well-being.

### Practical recommendations

Digital training has boomed in recent times and seems to persist in university education. The pandemic has shown that despite the difficulties, CGOL might have beneficial effects on students. Although face-to-face university teaching should not be substituted, our study shows that the use of digital resources for online training contributes to improving students’ positive emotions. For this reason, universities might consider maintaining or at least increasing the online or blended teaching model (face-to-face and online). In a digital world in which university students are considered digital natives, the use of these methodologies fits with their way of conceiving teaching.

The insights also provide support for online learning in the post-pandemic era, and more specifically, for generating positive emotions. We find that attitude is the main predictor of using CGOL resources. Contrary to what might be expected for first-year university students, attitude influences the participation in CGOL methodologies even more than social interaction. This finding might help teachers prioritise perceived usefulness, entertainment, and habit as key drivers to enhance students´ attitude towards these methodologies.

### Limitations and future research

This study makes use of concrete data from first-year students at the School of Economics and Business Sciences and the School of Law at a specific university. It would be advisable to replicate the study at other universities, and to make use of longitudinal data with the aim of detecting possible temporal effects. Furthermore, this study was geared at making a first attempt to analyse the holistic CGOL effect on positive emotions, independently of the subject studied and the specific CGOL used or the metacognise awareness of the team members. Future studies might expand this knowledge in connection with specific CGOL activities and concrete emotional effects, learning effects and performance effects. In addition, future studies could focus on the process of applying CGOL activities, describing the main phases and defining each phase content.

The conclusions should be interpreted with caution, as they might lead to different results in other settings, cultures and age groups (secondary education or even lifelong learning courses). Moreover, learning-related emotions are not considered in the present study and the robustness of the model was not checked. It would also be challenging to analyse whether the methodologies applied might also have effects on learning emotions.

Finally, it would be inspiring to study whether drivers other than those included here might have effects on attitude or on other constructs that differ from the ones studied in this paper, such as effort, stress or academic performance.

## Data Availability

The datasets generated during and/or analysed during the current study are available from the corresponding author on reasonable request.
